# *Rickettsia parkeri* in Brazil

**DOI:** 10.3201/eid1307.061397

**Published:** 2007-07

**Authors:** Iara Silveira, Richard C. Pacheco, Matias P. J. Szabó, Hernani G. C. Ramos, Marcelo B. Labruna

**Affiliations:** * University of São Paulo, São Paulo, Brazil; † Federal University of Uberlândia, Uberlândia, Minas Gerais, Brazil

**Keywords:** Rickettsia parkeri, Amblyomma triste, tick, isolation, Brazil, dispatch

## Abstract

We report finding *Rickettsia parkeri* in Brazil in 9.7% of *Amblyomma triste* ticks examined. An *R. parkeri* isolate was successfully established in Vero cell culture. Molecular characterization of the agent was performed by DNA sequencing of portions of the rickettsial genes *gltA, htrA, ompA,* and *ompB*.

The first reported infection with *Rickettsia parkeri* was in *Amblyomma maculatum* ticks in Texas >65 years ago (*1*). Although its pathogenicity for humans was suspected or speculated during the following decades (*2*), *R. parkeri* was only recently recognized as a human tickborne pathogen (*3*). Extensive cross-reactivity exists among spotted fever group rickettsiae—especially *R. rickettsii* (the etiologic agent of Rocky Mountain spotted fever [RMSF] and Brazilian spotted fever [BSF])—and *R. parkeri.* Most of the time, *R. rickettsii* antigen is the only antigen used in serologic analysis for routine diagnosis of RMSF and BSF. Thus, many human cases of *R. parkeri* infection may be routinely misidentified as RMSF (*2*).

During the 1990s in Uruguay, several human cases of a tickborne rickettsiosis were diagnosed on the basis of serologic analyses; the spotted fever group organism *R. conorii* was used as antigen (*4*). Because *R. conorii* has never been found in the Western Hemisphere, another spotted fever group rickettsia may have been responsible for the reported cases (*5*). Because of recent reports of *R. parkeri* infection among *A. triste* ticks in Uruguay (where *A. triste* is the most common human-biting tick), this rickettsia has been suggested as the most probable agent of the Uruguayan spotted fever rickettsiosis (*5*,*6*). These data are corroborated by similar clinical findings found for both the American spotted fever caused by *R. parkeri* and Uruguayan spotted fever (*2*,*4*). *R. parkeri* has been reported only in the United States and Uruguay. We report *R. parkeri* infection of *A. triste* ticks in Brazil.

## The Study

*A. triste* ticks were collected in a marsh area (21°07′06.7′′S, 51°46′06.5′′W) in Paulicéia County, state of São Paulo, Brazil. This area harbors a natural population of *A. triste,* mostly in the natural marsh environment along the Paraná River (*7*). Marsh deer (*Blastocerus dichotomus*) have been implicated as primary hosts for the adult stage of *A. triste* in the area, but the hosts for the immature stages of the tick remain unknown (*7*).

In January 2005, free-living adult *A. triste* ticks were collected by use of dry ice traps. Collected ticks were taken alive to the laboratory, where they were screened for rickettsial infection by using the hemolymph test with Gimenez staining (*8*). Immediately after hemolymph was collected, the ticks were stored at –80°C until used for further testing.

Ticks with hemolymph test results positive for infection with a *Rickettsia-*like organism were processed for isolation of *Rickettsia* in cell culture by using the shell vial technique (*9*). In brief, Vero cells were inoculated with tick body homogenate and incubated at 28°C. The level of cell infection was monitored by Gimenez staining of scraped cells from the inoculated monolayer; a rickettsial isolate was considered established after 3 passages, each reaching >90% of infected cells (*9*).

For cell isolation, a sample of 100%-infected cells from the fourth Vero cell passage was subjected to DNA extraction and thereafter tested by a battery of PCRs by using previously described primer pairs that targeted fragments of the rickettsial genes *gltA, htrA, ompA,* and *ompB* (*10*). Amplified products were purified and sequenced (*9*) and then compared with National Center for Biotechnology Information (NCBI) nucleotide BLAST searches (www.ncbi.nlm.nih.gov/blast).

Tick specimens with hemolymph test results negative for *Rickettsia*-like organisms were thawed and individually processed for DNA extraction by the guanidine isothiocyanate–phenol technique (*11*). PCR amplification of a rickettsial gene fragment (398 nt) of the citrate synthase gene (*gltA*) was attempted on DNA from each tick by using the primers CS-78 and CS-323, which were designed to amplify DNA from all known *Rickettsia* spp. (*9*). Tick samples shown by PCR to be positive were tested further by a second PCR, which used the primers Rr190.70p and Rr190.602n, which amplify a 530-nt fragment of most of the spotted fever group *Rickettsia* (*12*). PCR products of the expected sizes were purified and sequenced (*9*) and then compared with NCBI nucleotide BLAST searches.

A total of 31 adult specimens of *A. triste* ticks were collected in January 2005. Specimens from 3 of the 31 ticks contained *Rickettsia*-like organisms, as determined by the hemolymph test. PCR amplification of the remaining 28 tick specimens was negative for *Rickettsia* spp. A *Rickettsia* organism was successfully isolated from only 1 of the 3 ticks with positive hemolymph test results. The isolate, designated as At24, was successfully established in Vero cell culture. PCR performed on DNA extracted from infected cells yielded the expected PCR products for all reactions. After DNA sequencing, the generated sequences of 1093, 489, 479, and 775 nt for the *gltA, htrA, ompA,* and *ompB* genes, respectively, showed 100%, 99.8%, 100%, and 100% identity to corresponding sequences of *R. parkeri* Maculatum strain from the United States (GenBank accession nos. U59732, U17008, U43802, AF123717, respectively). Isolation attempts for the other 2 ticks with positive hemolymph test results were lost because of bacterial or fungal contamination. Nevertheless, remnants of ticks used to inoculate Vero cells were subjected to DNA extraction and tested by PCR for the *gltA* and *ompA* genes, as described above for ticks. Expected products were obtained from these PCR studies, and the generated sequences were 100% identical to the corresponding sequences of *R. parkeri* Maculatum strain (GenBank accession nos. U59732 and U43802, respectively). The frequency of *R. parkeri* infection among ticks examined in this study was 9.7% (3/31). Partial sequences (*gltA, htrA, ompA, ompB*) from *R. parkeri* strain At24 generated in this study were deposited into GenBank and assigned nucleotide accession nos. EF102236–EF102239, respectively.

## Conclusions

Our report of *R. parkeri* infection of ≈10% of *A. triste* ticks from 1 area in the state of São Paulo highlights the possibility of *R. parkeri* causing human cases of spotted fever rickettsiosis in Brazil. However, in contrast to Uruguay, Brazil appears to have rare occurrences of *A. triste* and has never had a report of an *A. triste* bite in humans. In addition, no human case of spotted fever has been reported from sites within the known distribution area of *A. triste* in Brazil. On the other hand, an *R. parkeri*–like agent (strain Cooperi) was recently reported to have infected *A. dubitatum* ticks from a BSF-endemic area in São Paulo (*9*). Since *A. dubitatum* is a human-biting tick that is highly prevalent in many BSF-endemic areas (*13*), it is a potential candidate for transmission of *R. parkeri* to humans.

Spotted fevers caused by *R. parkeri* and by *R. rickettsii* differ in 2 ways: an eschar frequently occurs at the tick bite site in spotted fever cases caused by *R. parkeri*, and lymphadenopathy occurs in cases caused by *R. parkeri.* Because clinical descriptions of BSF (diagnosed solely by serologic testing that uses *R. rickettsii* antigen) with these specific clinical signs have been described recently in Brazil (*14*,*15*), human infections with *R. parkeri* may be occurring in this country. These clinical descriptions were from areas with large populations of *A. dubitatum* but no known occurrence of *A. triste.* Moreover, because *R. rickettsii* antigen has been the only antigen regularly used for diagnosis of BSF, human spotted fever cases due to *R. parkeri* or other spotted fever group rickettsiae may be misidentified as BSF in Brazil.

Our study demonstrated an exact concordance between ticks that were positive for *Rickettsia*-like organisms by the hemolymph test and those that were positive for rickettsial DNA by PCR. Previous studies in our laboratory (*9*–*11*) have demonstrated the same results or a slightly higher sensitivity of PCR for detection of rickettsiae in ticks.
